# Stage-Specific Immune Responses to AgB T-Peptides in Patients with Cystic Echinococcosis

**DOI:** 10.3390/idr17030051

**Published:** 2025-05-07

**Authors:** Settimia Sbarra, Ambra Vola, Francesca Tamarozzi, Saeid Najafi-Fard, Alessandra Ludovisi, Antonella Teggi, Emanuele Nicastri, Fabrizio Albarello, Enrico Brunetti, Delia Goletti, Linda Petrone

**Affiliations:** 1 Translational Research Unit, National Institute for Infectious Diseases “Lazzaro Spallanzani”—IRCCS, 00149 Rome, Italy; 2 Microbiology and Virology Unit, IRCCS San Matteo Hospital Foundation, 27100 Pavia, Italy; 3 Department of Infectious Tropical Diseases and Microbiology, IRCCS Sacro Cuore Don Calabria Hospital, Negrar di Valpolicella, 37024 Verona, Italy; 4 Foodborne and Neglected Parasitoses Unit, Department of Infectious Diseases, Istituto Superiore di Sanità, 00161 Rome, Italy; 5 Department of Infectious and Tropical Diseases, Sant’Andrea Hospital University of Rome “Sapienza”, 00189 Rome, Italy; 6 Clinical Division of Infectious Diseases, National Institute for Infectious Diseases “Lazzaro Spallanzani”—IRCCS, 00149 Rome, Italy; 7 Diagnostic Imaging of Infectious Diseases, National Institute for Infectious Diseases “Lazzaro Spallanzani”—IRCCS, 00149 Rome, Italy; 8 Unit of Infectious Diseases, IRCCS San Matteo Hospital Foundation, 27100 Pavia, Italy; 9 Department of Clinical, Surgical Diagnostic and Pediatric Sciences, University of Pavia, 27100 Pavia, Italy; 10 WHO Collaborating Centre for Clinical Management of Cystic Echinococcosis, University of Pavia, 27100 Pavia, Italy

**Keywords:** human cystic echinococcosis, peptides, IL-4, antigen B, whole blood, transitional stage cysts, ultrasonography, IL-7

## Abstract

**Background:** The identification of parasite- and stage-specific antigens is crucial for the development of new diagnostic tests for cystic echinococcosis (CE). We previously analysed the interleukin (IL)-4 response to T-specific peptides corresponding to the immunogenic regions of the five antigen B (AgB) subunits, demonstrating that AgB1 is the most immunogenic protein and that the response to all AgB peptides is associated with viable cysts. However, the response in patients with CE3a (WHO-IWGE) cystic stage was not evaluated and no other immunological factors besides IL-4 were included in the analysis. **Methods:** Four study groups were defined: “CE3a group” (transitional cysts), “CE3b group” (active cysts), “CE4/CE5 group” (inactive cysts), and “NO CE-group” encompassing patients with non-CE cysts (controls). Whole blood was stimulated in vitro with the five different T-specific peptide pools corresponding to the five AgB subunits and with a pool containing all five peptides’ pools (total pool). IL-4 and other immunological markers were evaluated by ELISA and a multiplex assay, respectively. **Results:** Twenty-four patients with CE (CE3a-group n = 3; CE3b-group n = 6; CE4/CE5-group n = 15) and 14 subjects with non-CE cysts were enrolled. IL-4 levels in response to AgB1 and AgB3 pools were significantly increased in CE compared to NO CE groups (*p* = 0.0201, *p* = 0.0041). Within the CE patients, the highest IL-4 median level was observed in response to the AgB total pool, the AgB3 and AgB4 pools, followed by the AgB1 pool. Moreover, the IL-4 levels in response to the AgB1 pool were found to be significantly higher in the CE3b group compared to the CE4/CE5 group (*p* = 0.0070), while no differences were found for the CE3a group. As for other cytokines, we found higher IL-7 levels in response to the AgB4 pool in the CE4/CE5 group compared to the CE3b group (*p* = 0.0012), higher IL-2 levels in response to the AgB1 pool and AgB total pool in CE3b patients compared to controls (*p* = 0.0016), and higher IL-13 levels in response to the AgB total pool in patients with CE3b and CE4/CE5 cysts compared to NO CE (*p* = 0.0016; *p* = 0.0009). **Conclusions:** These results contribute to a better knowledge of the immune interplay in the presence of CE and may be useful for further exploring the use of recombinant proteins/peptides in cytokine release assays for the diagnosis and follow-up of CE.

## 1. Introduction

**Figure 1:**
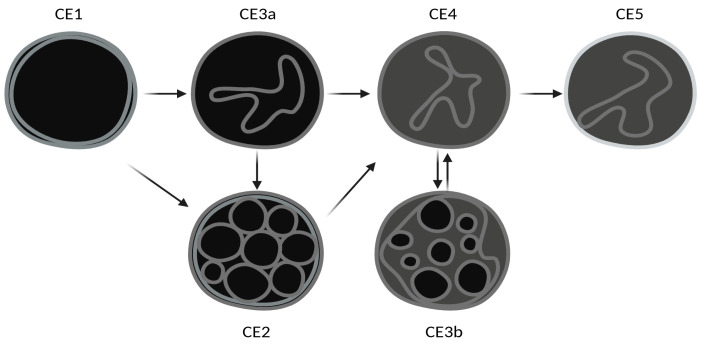
Representation of possible CE cyst progression. Created with Biorender.com.

Cystic echinococcosis (CE) is a widespread parasitic zoonosis caused by *Echinococcus granulosus* sensu lato [1]. Nine genotypes (G1-G8 and G10) and five species are described: *E. granulosus* sensu stricto (s.s.) (G1-G3), *E. equinus* (G4), *E. ortleppi* (G5), *E. canadensis* (G6-G8 and G10), and *E. felidis* [2,3]. Most human infections are caused by *E. granulosus* s.s. The life cycle of all *Echinococcus* species encompasses the development of the parasite adult sexual stage in the intestine of a definitive host, usually carnivorous, which releases infective eggs with feces, and of a larval stage (metacestode) in tissues of an intermediate host, mostly non-carnivorous, which become infected through the ingestion of parasite eggs. The cycle completes when the definitive host feeds on organs of an intermediate host containing a fertile metacestode [4]. The definitive host of *E. granulosus* s.l. is the dog (or other canids), while intermediate hosts are livestock or wild ungulates, where the metacestode develops in the form of a concentrically expanding, well-defined cyst [5]. Humans are accidental intermediate hosts, where the ingestion of *E. granulosus* eggs might cause the development of the larval cystic form, mainly in the liver, which can lead to severe complications [6]. Besides *E. granulosus* sensu latu, *E. multilocularis*, the etiological agent of alveolar echinococcosis (AE) [7,8], is a considerable public health problem in the Northern Hemisphere, while neotropical echinococcosis caused by E. oligarthra and E. voeli, limited to South America, only rarely occurs in humans. Although belonging to the same genus, *E. granulosus*, *E. multilocularis*, and the other rare species cause very different diseases [8,9,10]. The diagnosis of CE is based on imaging, especially ultrasonography (US). *E. granulosus* cysts are classified according to the WHO-Informal Working Group on Echinococcosis (WHO-IWGE) US-based classification into six stages, in turn grouped into “active” (CE1, CE2), “transitional” (CE3a and CE3b), and “inactive” (CE4 and CE5) from their natural history perspective [11]. Indeed, CE3a and CE3b cysts are referred to as “transitional” cysts from an evolutionary perspective along the natural history of CE (CE3a being a “transitional” stage between CE1 and CE4 and CE3b being a reactivation from a CE4 stage), but their viability profile is different (Figure 1). Correspondence between US staging and viability is, indeed, not perfect, since CE1, CE2, and CE3b cysts are viable; CE3a cysts have the same probability of being viable or non-viable; and CE4 and CE5 cysts are considered non-viable if the stage has been spontaneously reached, but a variable proportion of CE4 might still be biologically viable, especially if inactivation is reached through treatment [12,13,14]. For CE3a and CE4 stages, biological viability can only be assessed by the analysis of invasively collected cyst material (biopsy, surgery), since no biological marker, including serology, is available that could reliably indicate CE cyst viability [15]. Thus, while classifying CE cysts is crucial for managing the disease correctly, long-term follow-up with US to detect changes in cyst morphology (i.e., change in stage) is required to evaluate the outcome of the applied clinical management approach in terms of biological cure [11]. In this context, the identification of parasite- and stage-specific antigens, and/or immunological responses to them, is an initial crucial step for the development of new immunodiagnostic tests for the diagnosis and follow-up of CE. Furthermore, the analysis of the immune response to different antigens could contribute to an understanding of the host–pathogen interplay resulting in the presence of different cyst stages [16]. Among *E. granulosus* antigens, Antigen B (AgB), one of the most abundant antigens of the CE cyst fluid, has been extensively studied. It is highly immunogenic in human infections and has high sensitivity and specificity in serological tests [17].

Antigen B (AgB) is encoded by a multigene family encoding five antigenic subunits (AgB1-AgB5) [18,19]. The IgG reactivity of all AgB subunits for CE diagnosis has been evaluated, and each subunit shows a different reactivity, with AgB1 as the highest immunogenic protein [20]. However, the AgB subunits could be differentially expressed within single cysts and/or throughout the parasite developmental stages or strains [21].

We previously analysed the interleukin (IL)-4 response to T-specific peptides corresponding to the immunogenic regions of the five AgB subunits, demonstrating that AgB1 is the most immunogenic protein and that the response to all AgB peptides is associated with viable cysts in our experimental setting [22]. However, the response in CE3a cystic stages was not evaluated and no other immunological factors besides IL-4 were included in the analysis. Considering the substantial biological differences between these stages, as described above, in this study, we evaluated the T-specific response to the AgB peptides in patients with CE3a (unknown viability), CE3b (viable), and CE4/CE5 cysts (considered non-viable based on inclusion criteria, see below) in terms of IL-4 production and other immunological factors to better characterize the immune response in these stages and potentially identify immunological biomarkers associated with parasite viability.

## 2. Materials and Methods

### 2.1. Ethics Statement

The Ethics Committees of IRCCS San Matteo Hospital Foundation, (Pavia, Italy), Istituto Nazionale per le Malattie Infettive (INMI) “Lazzaro Spallanzani”—IRCCS (Roma, Italy), and of Sant’Andrea Hospital (Rome, Italy) approved the study (approval numbers: P-20180022622; 28/2014, 59/2014, 16/2018, 146/2020, 34/2010, 580/11). Written informed consent was obtained to participate in the study.

### 2.2. Enrollment

The study included patients with hepatic CE cysts and “NO CE” control subjects who attended the outpatient clinic of IRCCS San Matteo Hospital Foundation, INMI, and Sant’Andrea Hospital.

CE patients were diagnosed based on the presence of a CE cyst in the liver identified by ultrasound (or other imaging techniques, e.g., computed tomography or magnetic resonance) and the presence of pathognomonic features. “NO-CE” subjects were patients with focal liver lesions suspected of CE but who had the CE diagnosis excluded by imaging and with a negative serology.

Cysts were staged according to the WHO-IWGE classification and only patients with CE3a, CE3b, CE4, and CE5 were enrolled. Patients in the “CE4/CE5-group” had ≥1 CE4 or CE5 cyst, either from spontaneous inactivation or from treatment, provided the latter had been concluded at least four years before enrollment. This criterion was adopted as the vast majority of treatment-induced CE4 reactivate within two years from the end of treatment itself [11]; therefore, these cysts could be considered bona fide not viable. No restriction relative to previous treatment history was applied to patients in the CE3a and CE3b groups. Four study groups were defined: “CE3a-group”, “CE3b-group”, “CE4/CE5 group”, and “NO CE-group”.

The exclusion criteria for all the study groups were the presence of extra-hepatic cysts or the presence of >1 active hepatic cyst in the CE3a or CE4/CE5 groups or in different active stages in the CE3b group.

Patients with multiple cysts who fulfilled inclusion and exclusion criteria were classified according to the cyst having the more active stage (i.e. CE3b > CE3a > CE4/CE5).

### 2.3. AgB Peptides

AgB synthetic peptides were designed and used as previously described [22]. Peptides corresponding to the same AgB subunit were pooled. Five pools were obtained, named from AgB1 pool to AgB5 pool. Furthermore, a “total pool” was obtained by pooling all the peptides.

### 2.4. Whole Blood Assay

As described before [23], heparinized whole blood (0.5 mL per well) was stimulated with peptide pools at 1 or 10 ug/mL, an enriched fraction of native AgB at 1–4 ug/mL as a control antigen, and staphylococcal enterotoxin B (SEB) (Sigma, St Louis, MO, USA) at 200 ng/mL as a positive control. Unstimulated samples were included as negative controls. Stimulated or unstimulated whole blood samples were incubated overnight at 37 °C, 5% CO_2_. Supernatants were then harvested and frozen until analysis.

### 2.5. Cytokine Analysis

Interleukin-4 levels in response to AgB peptide pools were evaluated by a highly sensitive ELISA (Quantikine HS IL-4 ELISA, R&D Systems, Minneapolis, MN, USA), following the manufacturer’s instructions. Range of detection was 0.25–16 pg/mL. Stimulated plasma samples were also tested for a panel of cytokines, chemokines, and growth factors, included in the Bio-Plex Pro Human Cytokine 27-plex Assay panel (Bio-Rad, Hercules, CA, USA). The panel comprises: IL-1β, IL-1RA, IL-2, IL-5, IL-6, IL-7, IL-8, IL-9, IL-10, IL-12p70, IL-13, IL-15, IL-17A, eotaxin, basic fibroblast growth factor (FGF), granulocyte colony-stimulating factor (G-CSF), granulocyte-macrophage colony-stimulating factor (GM-CSF), Interferon (IFN)-γ, IFN-γ induced protein 10 (IP-10), monocyte chemoattractant protein 1 (MCP-1), macrophage inflammatory protein (MIP)-1α, MIP-1β, platelet-derived growth factor (PDGF), RANTES (regulated on activation, normal T cell expressed and secreted), tumor necrosis factor-α (TNF-α), and vascular endothelial growth factor (VEGF). The Bioplex200 system (Bio-Rad, Hercules, CA, USA) instrument was used as a reader. Values below the lower detection limit were converted to 0, whereas values above the upper detection limit were converted to the highest value of the standard curve. Moreover, only values originating from ≥50 bead readings were considered for the analysis. For all the analytes evaluated, the values of each sample were subtracted from those of the unstimulated control.

### 2.6. Statistical Analysis

Prism 8 software (Graphpad Software 8.0, San Diego, MO, USA) was used for data graphing and statistical analysis. Medians, interquartile ranges (IQRs), or range (min–max) were calculated for continuous measures. The Chi-square test was applied for dichotomous measures. The Wilcoxon and Mann–Whitney U tests (for paired or unpaired data, respectively) were used for pairwise comparison, while the Kruskal–Wallis test was used for comparison among groups. Statistical significance was set at *p* < 0.05 or at *p*-values derived from Bonferroni correction.

## 3. Results

### 3.1. Study Population

We enrolled 24 patients with CE and 14 control subjects between November 2016 and February 2020.

Based on the inclusion and exclusion criteria, three CE patients (12.5%) were included in the CE3a group, six (25%) were included in the CE3b group, and fifteen (62.5%) were included in the CE4/CE5 group. Clinical and demographic characteristics are reported in Table 1. In the CE3a group, two patients (2/3 patients, 67%) reported previous treatment. In the CE3b group, three patients (3/6 patients, 50%) reported a previous treatment. Within the CE4/CE5 group, four patients (4/15, 27%) had received prior pharmacological treatment, which terminated at least 4 years before their enrolment. At the time of sample collection, one patient (1/3 patients, 33%) with a CE3a cyst and two patients (2/6 patients, 33%) with CE3b cysts were undergoing treatment.

NO CE patients were diagnosed with cystadenoma, simple liver cysts, serous cysts, or non-parasitic cysts.

### 3.2. The IL-4 Response to AgB1 and to AgB3 Pools Is Associated with CE

**Figure 2:**
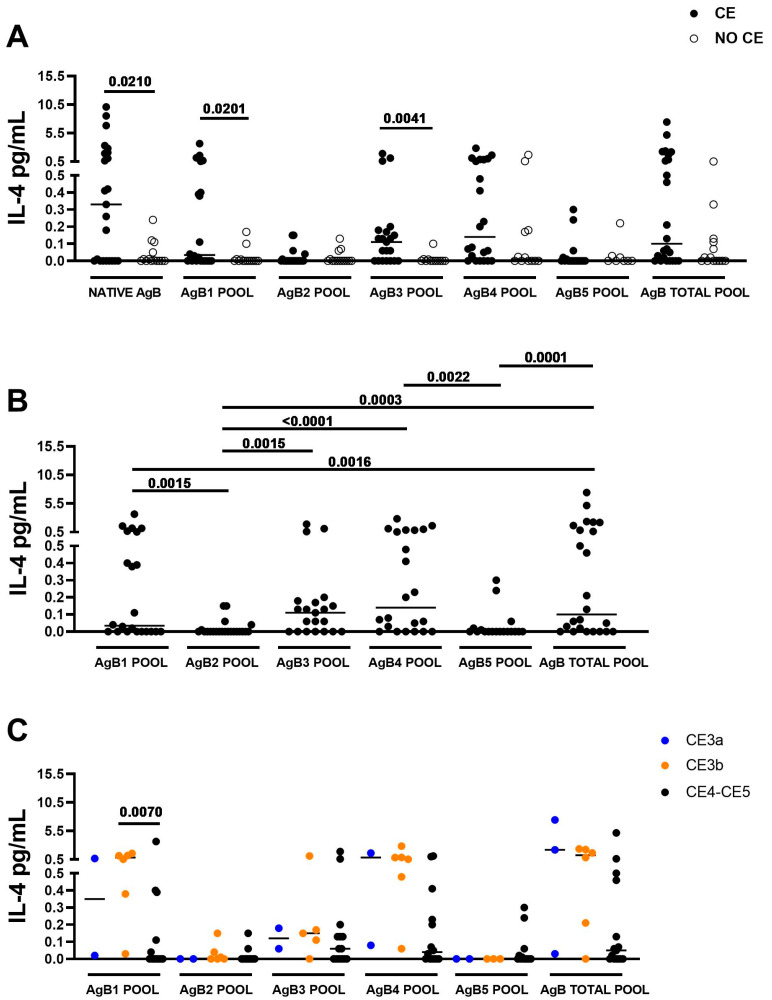
The IL-4-specific response to AgB and peptide pools corresponding to the AgB1 and AgB3 subunits is associated with CE. (**A**) IL-4 levels in response to AgB1 and AgB3 pools were significantly increased in CE compared to NO CE (*p* = 0.0201, *p* = 0.0041). (**B**) AgB3 and AgB4, followed by the AgB1 pool, are the most immunogenic subunits in CE patients (at least *p* = 0.0022). (**C**) IL-4 levels in response to the AgB1 pool are significantly higher in the CE3b group than in the CE4/CE5 group (*p* = 0.0070). Medians are shown. Wilcoxon U and Mann–Whitney tests were used to compare groups. Statistical significance was set at *p* < 0.05 or *p* resulting from Bonferroni correction. Footnotes: AgB: Antigen B; IL: Interleukin; CE: cystic echinococcosis.

When assessing the IL-4 response to the different AgB peptide pools in the enrolled subjects, the intergroup analysis showed that median IL-4 production was significantly higher in CE patients compared to NO CE subjects after whole blood stimulation with the AgB1 and AgB3 pools (*p* = 0.0201; *p* = 0.0041). Moreover, as previously found [22,23,24], the whole blood IL-4 response to the native AgB and the total AgB pool was associated with CE infection (*p* = 0.0210 and *p* = 0.0525, respectively) (Figure 2A).

No significant differences in median IL-4 response to AgB2, AgB4, and AgB5 pools were observed between CE and NO CE subjects (Figure 2A).

The intragroup analysis within different CE groups showed that the highest IL-4 responses were observed after stimulating the whole blood with the total pool, the AgB3 and AgB4 pools, followed by the AgB1 pool (*p* ≤ 0.0022 in all comparisons) (Figure 2B). Moreover, the median IL-4 level induced by the AgB total pool was comparable with those induced by the AgB3 and AgB4 pools.

### 3.3. The IL-4 Levels in Response to AgB1 Pool Are Significantly Higher in Presence of CE3b Cyst

To identify differences in the IL-4 response to AgB in patients with CE cysts in different viability conditions, we stratified them into the CE3a group (n = 3), the CE3b group (n = 4), and the CE4/CE5 group (n = 15).

Median IL-4 levels were significantly higher in the CE3b group compared to the CE4/CE5 group in response to the AgB1 pool (*p* = 0.0070).

Although not significant, higher median IL-4 levels were observed in patients with CE3b cysts compared to patients with CE4/CE5 cysts also in response to the AgB4 pool (*p* = 0.0179) and AgB total pool (*p* = 0.0503) (Figure 2C).

Moreover, even if only three subjects were evaluated, and the median IL-4 values did not differ with those of the CE3b group, it can be noticed (Figure 2C) that the production of IL-4 after stimulation with the AgB1, AgB4, and AgB total pools differed among single individuals with either high or very low levels.

### 3.4. IL-7 Response Is Associated with CE4/CE5 Cysts

**Figure 3:**
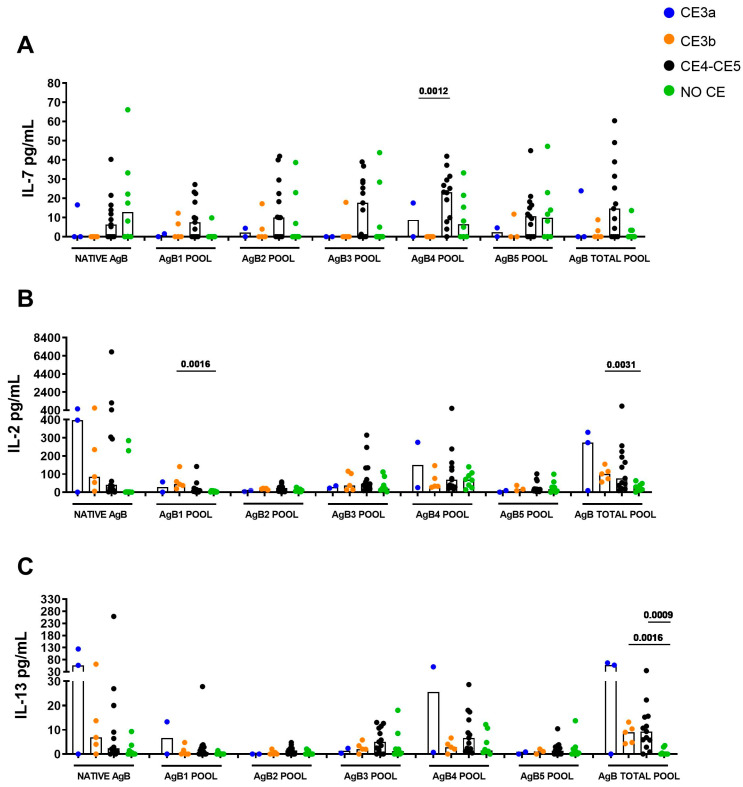
Evaluation of immunological factors in response to AgB peptide pools. (**A**) IL-7 levels in response to the AgB4 pool were significantly elevated in patients with CE4/CE5 cysts compared to patients with CE3b cysts (*p* = 0.0012). (**B**) IL-2 levels in response to the AgB1 pool and total AgB pool were significantly increased in CE3b patients compared to controls (*p* = 0.0016), (*p* = 0.0031). (**C**) IL-13 levels in response to the AgB total pool were significantly increased in patients with CE3b cysts and CE4/CE5 cysts compared to NO CE (*p* = 0.0016), (*p* = 0.0009). Medians are shown. The Mann–Whitney test was used to compare groups. Statistical significance was set at *p* < 0.05 or *p* resulting from Bonferroni correction. Footnotes: AgB: Antigen B; IL: Interleukin; CE: cystic echinococcosis.

To assess whether other immune factors can be induced by the different AgB peptides in a stage-specific manner, we tested a panel of 27 cytokines, chemokines, or growth factors in plasma stimulated with native AgB and the different AgB peptide pools. The results are reported in Table S1 and summarized in Figures S1–S3. Interestingly, in the CE4/CE5 group, higher median IL-7 levels were found in response to the AgB4 pool compared to patients with CE3b cysts (*p* = 0.0012) but not CE3a cysts (Figure 3A). Finally, we observed higher median IL-2 levels in CE3b patients compared to controls in response to the AgB1 pool (median 43.80 pg/mL, IQR: 28.10–100.1 vs. 2.74 pg/mL, IQR: 1.1–7.5) and AgB total pool (median 101.2 pg/mL, IQR: 65.1–134.6 vs. 22.9 pg/mL, IQR: 11.1–45.9) (*p* = 0.0016 and *p*= 0.0031, respectively), and a higher IL-13 level in response to the AgB total pool in patients with CE3b and CE4/CE5 cysts compared to NO CE subjects (*p* = 0.0016, median 9.00 pg/mL, IQR: 4.5–11.8 vs. 0 pg/mL, IQR: 0–2.3; *p* = 0.0009,median 9.3 pg/mL, IQR: 4.3–15.5 vs. 0 pg/mL, IQR: 0–2.3) (Figure 3B,C).

## 4. Discussion

In the absence of a diagnostic biomarker able to etiologically diagnose CE cysts and their viability status, imaging (primarily US, supported by serology and/or contrast-enhanced imaging and biopsy) is relied upon for the diagnosis of infection and long-term follow-up with US to detect changes in cyst morphology (i.e., change in stage) to evaluate the outcome of the applied clinical management approach in terms of biological cure. Therefore, the identification of parasite- and stage-specific antigens and/or the immunological response to them, is an initial crucial step for the development of new immunodiagnostic tests for the diagnosis and follow-up of CE. Moreover, the development of an assay based on peptides or recombinant antigens could also be particularly appealing to overcome the problems related to the use of native antigens (e.g., variability, procurement, scalability) [22]. In this study, confirming our previous findings [22], we found that the AgB1 and AgB3 pools, AgB total pool, and native AgB induced a high level of IL-4 in stimulated blood from CE patients compared to controls; moreover, we found that the response to AgB1 pool was associated with the CE3b stage. This result is paralleled by previous observations demonstrating the AgB1 subunit to be the most reactive protein when used to evaluate antibody responses, in particular, in patients with active cysts [20,25]. Therefore, being able to induce both a T and B cell response, AgB1 is suitable for developing both serological or T-cell based tests and could be useful in discriminating viable and non-viable cysts. The AgB3 subunit has been described as a low immunogenic protein due to its structure and high resistance to protease lysis [26], which may be translated into antigen processing difficulties by immune cells [18]. The use of peptides in this study may have overcome this issue by facilitating the recognition of linear epitopes in our experimental whole blood assay.

We also showed that additional factors, including IL-7, IL-2, and IL-13, are modulated in response to the AgB peptide pools. In particular, we observed significantly higher levels of IL-7 in response to AgB4 in patients with CE4/CE5 cysts (bona fide non-viable) compared to patients with CE3b (viable) cysts. Moreover, even if the differences were not statistically significant, IL-7 levels were higher in the CE4/CE5 group in response to all the AgB subunits, suggesting a role of this cytokine in the presence of inactive CE. This cytokine is important for the development and the survival of T cells, including memory T cells [27]. However, the IL-7 involvement during CE has not been investigated yet. IL-7, and other growth factors like FGF, was shown to be increased in patients treated for strongyloidiasis [28], suggesting its involvement in parasite elimination. Despite the significant differences between strongyloidiasis and cystic echinococcosis, it is plausible that the parasite is no longer viable in the evaluated CE4/CE5 cysts group [12]. This suggests a potential role of IL-7 in restoring tissue integrity and homeostasis during parasite containment. Future studies, investigating IL-7 at the site of cyst development [29], may be useful to address this issue.

We found increased levels of IL-2, a key factor for T-cell survival, differentiation [30], and Th1 responses, in response to the AgB1 subunit pool and to the total AgB pool in patients with CE3b cysts compared to the controls. This result is in line with previous findings suggesting the presence of a mixed Th1/Th2 response in T cell lines derived from patients with active and transitional cyst [31]. Moreover, we found that the polyfunctional CD4^+^ T cell subtype secreting IL-2, Th2 cytokines, and TNF-α associates with patients with cysts in active stages [32], confirming the ability of AgB (and its subunits) to induce both Th1 and Th2 cytokines. Nevertheless, it remains unclear whether the immune profile in patients with different CE stages is the cause or the consequence of the presence of that cyst stage. Moreover, they highlight that the nature of antigens recognized by the host’s immune system may be crucial in these immunoregulation mechanisms.

IL-13 levels were elevated in response to the total pool of AgB in patients with CE3b cysts and CE4/CE5 cysts compared to controls. Both IL-4 and IL-13 are considered markers of a Th2 response [33], and both cytokines were found in the serum of patients with CE3a or CE4/CE5 cysts [34], and in plasma exosomes from patients with active CE cysts [35]. Altogether, these findings, besides underlighting the already demonstrated induction of a Th2 response during CE [15,32,36], suggest the evaluation of IL-13 in addition to IL-4 for CE identification in a cytokine release assay, using a large sample size and the same analytical method.

This study has several limitations, including having a small sample size, particularly for the CE3a and CE3b groups, which, despite their good characterization, makes it difficult to derive strong conclusions from the study results. The observation that the production of IL-4 by blood from patients stimulated with the AgB1, AgB4, and AgB total pools differed among single individuals with CE3a and to some extent also with CE4/CE5, with either high or very low levels being produced, might suggest that the viability of the cysts within these ultrasonographically classified stages might differ in terms of biological viability. However, the cross-sectional design of the study hampered the identification of the real viability status of the single cysts.

Although we did not calculate the accuracy of AgB pool-stimulated IL-4 levels in identifying the different CE stages, our results suggest that this assay would not be suitable for clinical application on individual subjects. Another limitation of this study is the exclusive use of AgB subunit peptides. Studying both linear and conformational epitopes using proteins would have provided a more comprehensive characterization of the immune response to this complex antigen. However, the use of recombinant proteins in this study could not be implemented due to financial and time-related constraints for obtaining high-quality reagents.

In conclusion, here, we confirm that IL-4 levels in response to AgB1 were significantly higher in patients with CE compared to control patients with non-CE cysts and, within CE, in patients with CE3b compared to patients with CE4/CE5. Moreover, we found high IL-7 levels in the CE4/CE5 group in response to the AgB4 pool, and higher IL-13 levels in response to the AgB total pool in the CE group compared to controls. These results contribute to a better knowledge of the immune interplay in the presence of CE and may be useful for further exploring the use of recombinant proteins or peptides in cytokine release assays for the diagnosis and follow-up of CE.

## Figures and Tables

**Table 1:** Demographic and clinical characteristics of the enrolled subjects.

	CE			No CE	*p*
	CE3a	CE3b	CE4-CE5		
N (%)	3	6	15	14	
Median age in years (IQR)	36 (23–43)	51 (35–71)	51 (33–61)	65 (57–68)	0.0716 *
Female sex N (%)	3 (100)	1 (17)	9 (60)	12 (86)	**0.0136** ^#^
Serology positive results N (%) °	2 (67)	5 (83)	7 (47)	0 (0)	**0.0014**
Previous albendazole treatment N (%)	2 (67)	3 (50)	4 (27)	1 (7)	0.0748 ^#^
Current pharmacological treatment N (%)	1 (33)	2 (33)	0 (0)	1 (7)	0.0750
Cyst number/patient range	1–4	1–1	1–5	1–3 ^§^	0.3868 *

Footnote CE: cystic echinococcosis; N: number; IQR: interquartile range; ° Serology result refers to the result of the different assays routinely performed in the clinical centers. * Kruskal–Wallis; ^#^ Chi-square test; ^§^ subjects with multiple cysts were excluded. The bold values indicate statistically significant differences.

## Data Availability

The raw data generated and/or analyzed within the present study will be available in INMI institutional repository (rawdata.inmi.it), subject to registration. The data can be found by selecting the article of interest from a list of articles ordered by year of publication. No charge for granting access to data is required. In the event of a malfunction of the application, the request can be sent directly by e-mail to biblioteca@inmi.it.

## Supplementary Materials

The following supporting information can be downloaded at: https://www.mdpi.com/article/10.3390/idr17030051/s1, **Table S1**: Cytokines, chemokines, or growth factors measured in plasma stimulated with native AgB or the different AgB peptide pools. **Figure S1**: Evaluation of cytokines in response to AgB peptide pools in stimulated plasma samples by multiplex analysis. The values are reported as medians. The Mann–Whitney test was used to compare groups. Statistical significance was set at *p* < 0.05 or p resulting from Bonferroni correction. Footnotes: AgB: Antigen B; analytes concentrations were determined by multiplex assay. IL: interleukin; ra: receptor antagonist; IFN: Interferon; TNF: tumor necrosis factor. CE: cystic echinococcosis. **Figure S2:** Evaluation of chemokines in response to AgB peptide pools in stimulated plasma samples by multiplex analysis. The values are reported as medians. The Mann–Whitney test was used to compare groups. Statistical significance was set at *p* < 0.05 or p resulting from Bonferroni correction. Footnotes: AgB: Antigen B; analytes concentrations were determined by multiplex assay. IL: interleukin; IP: Interferon gamma-induced protein; MCP: monocyte chemoattractant protein; MIP: macrophage inflammatory protein; RANTES: regulated on activation, normal T cell expressed and secreted. CE: cystic echinococcosis. **Figure S3:** Evaluation of growth factors in response to AgB peptide pools in stimulated plasma samples by multiplex analysis. The values are reported as medians. The Mann–Whitney test was used to compare groups. Statistical significance was set at *p* < 0.05 or p resulting from Bonferroni correction. Footnotes: AgB: Antigen B; analytes concentrations were determined by multiplex assay. FGF: fibroblast growth factor; G-CSF: granulocyte-colony stimulating factor; GM-CSF: granulocyte-macrophage colony-stimulating factor; PDGF: platelet-derived growth factor; VEGF: vascular endothelial growth factor. CE: cystic echinococcosis.
