# Supplementary material for: Stage-Specific Immune Responses to AgB T-Peptides in Patients with Cystic Echinococcosis
Source: Infect Dis Rep. 2025 May 7;17(3):51. doi: 10.3390/idr17030051 (PMC12101248; doi:10.3390/idr17030051)
Supplement: Supplementary file 1 [file idr-17-00051-s001.zip › Table S1.pdf]

Table S1: Cytokines, chemokines, or growth factors measured in plasma stimulated with native AgB or the different AgB peptide pools.

**Function**	**Analyte**	**Median (IQR) pg/mL**			
		**AgB1 pool**			
		**CE3a**	**CE3b**	**CE4/CE5**	**CTR**
**Cytokines**	**IL-1β**	0.48 (0.00-0.96)	2.08 (0.56-6.88)	1.36 (0.08-6.42)	2.32 (1.52- 3.98)
	**IL1rα**	1212.00 (486.00-1937.00)	1023.00 (700.20-2733.00)	980.10 (348.90-1350.00	1142.00 (304.10-1623.00)
	**IL-2**	28.48 (0.00-56.96)	43.80 (28.10-100.10)	9.20 (3.82-19.04)	2.74 (1.09-7.49)
	**Il-4**	0.36 (0.12-0.60)	1.76 (0.62-8.18)	0.72 (0.14-2.32)	0.56 (0.05-0.89)
	**IL-5**	0.00 (0.00-0.00)	30.32 (11.56-118.90)	33.64 (1.98-61.20)	9.70(0.00-58.95)
	**IL-6**	26.60 (6.68-46.52)	16.32 (3.12-298.00)	34.52 (7.80-50.34)	8.46 (3.16-22.61)
	**IL-7**	0.78 (0.00-1.56)	0.00 (0.00-9.48)	7.52 (0.00-20.24)	0.00 (0.00-0.00)
	**IL-9**	10.28 (0.00-20.56)	35.72 (9.36-50.36)	21.12 (4.26-51.66)	0.42 (0.00-34.97)
	**IL-10**	0.56 (0.00-1.12)	2.12 (0.00-4.62)	1.32 (0.54-4.80)	1.00 (0.00-2.22)
	**IL-12**	2.32 (0.00-4.64)	0.00 (0.00-21.94)	6.40 (3.36-10.72)	3.56 (0.00-8.00)
	**IL-13**	6.66 (0.00-13.32)	0.00 (0.00-3.18)	0.80 (0.00-3.18)	0.00 (0.00-0.15)
	**IL-15**	0.00 (0.00-0.00)	74.00 (24.36-243.90)	48.72 (0.00-201.00)	83.36 (0.00-166.10)
	**IL-17**	2.30 (2.28-2.32)	5.20 (0.00-27.40)	4.08 (1.18-7.42)	1.28 (0.00-3.66)
	**IFN-γ**	33.40 (1.92-64.80)	39.50 (13.10-179.00)	46.60 (4.80-56.10)	16.30 (0.33-34.20)
	**TNF-α**	0.00 (0.00-0.00)	33.30 (5.02-58.80)	17.70 (7.82-24.60)	2.98 (0.00-8.40)
**Chemokines**	**IL-8**	119.60 (0.00-239.20)	776.40 (288.50-1072.00)	191.60 (15.20-928.80)	22.24 (0.00-197.20)
	**Eotaxin**	1.10 (0.00-2.20)	3.88 (0.36-11.08)	1.80 (0.00-11.14)	0.18 (0.00-4.60)
	**IP-10**	64.80 (22.30-107.00)	31.80 (0.00-1617.00)	456.00 (252.00-874.00)	176.00 (131.00-327.00)
	**MCP-1**	511.00 (4.12-1019.00)	563.00 (54.10-962.00)	523.00 (81.60-1424.00)	77.00 (7.66-285.00)
	**MIP-1α**	3.74 (1.08-6.40)	9.80 (3.24-201.00)	12.00 (3.32-17.50)	2.30 (0.60-6.57)
	**MIP-1β**	241.00 (31.10-452.00)	240.00 (96.90-1209.00)	349.00 (92.50-565.00)	121.00 (45.50-228.00)
	**RANTES**	59.20 (0.00-118.00)	424.00 (53.70-698.00)	301.00 (0.00-690.00)	0.00 (0.00-0.00)
**Growth factors**	**FGF**	5.14 (0.00-10.28)	15.48 (5.06-39.38)	10.36 (3.94-18.48)	3.06 (0.68-5.02)
	**G-CSF**	174.00 (9.80-338.00)	496.00 (164.00-6180.00)	467.00 (114.00-783.00)	119.00 (43.40-332.00)
	**GM-CSF**	0.60 (0.00-1.20)	3.64 (2.90-8.56)	2.08 (0.68-4.46)	0.00 (0.00-1.38)
	**VEGF**	44.40 (0.00-88.70)	72.80 (24.60-174.00)	0.00 (0.00-210.00)	66.00 (5.24-179.00)
	**PDGF**	0.00 (0.00-0.00)	0.00 (0.00-65.60)	6.76 (0.00-39.30)	2.60 (0.00-21.30)

Function	Analyte	Median (IQR) pg/mL			
		AgB2 pool			
		**CE3a**	**CE3b**	**CE4/CE5**	**CTR**
**Cytokines**	**IL-1β**	22.12 (20.72-23.52)	22.20 (8.30-39.78)	19.68 (14.30-73.74)	21.58 (1.05-53.18)
	**IL1rα**	7060.00 (6234.00-7886.00)	6371.00 (2165.00-7715.00)	3790.00 (1858.00-6388.00)	4791.00 (2611.00-6525.00)
	**IL-2**	6.40 (2.96-9.84)	20.24 (14.94-20.92)	23.76 (4.58-29.64)	13.16 (3.85-16.55)
	**Il-4**	3.50 (2.72-4.28)	8.84 (4.60-11.44)	5.60 (2.82-10.32)	5.02 (1.86-10.64)
	**IL-5**	37.58 (13.32-61.84)	130.00 (84.24-167.10)	143.50 (63.70-236.30)	74.10 (26.68-173.8)
	**IL-6**	426.50 (163.80-689.20)	548.50 (275.30-929.00)	457.20 (300.80-823.40)	303.50 (142.60-390.50)
	**IL-7**	2.20 (0.00-4.40)	0.00 (0.00-10.56)	9.96 (0.00-26.02)	0.00 (0.00-18.95)
	**IL-9**	18.24 (0.00-36.48)	63.68 (23.58-135.90)	65.68 (0.00-125.70)	25.74 (0.00-60.46)
	**IL-10**	9.34 (6.00-12.68)	5.16 (1.04-6.80)	10.00 (4.18-15.46)	8.14 (4.17-11.99)
	**IL-12**	2.32 (0.00-4.64)	8.84 (3.58-17.94)	11.96 (3.60-29.64)	11.90 (5.09-20.04)
	**IL-13**	0.00 (0.00-0.00)	0.00 (0.00-1.36)	1.48 (0.00-2.38)	0.00 (0.00-1.08)
	**IL-15**	266.60 (179.00-354.20)	251.70 (101.90-352.40)	380.00 (71.16-492.50)	222.90 (6.49-394.10)
	**IL-17**	16.68 (16.00-17.36)	35.16 (18.02-42.92)	25.68 (9.72-50.68)	19.86 (9.37-28.53)
	**IFN-γ**	118.00 (103.00-132.00)	82.40 (64.80-159.00)	103.00 (72.10-135.00)	87.30 (74.70-173.00)
	**TNF-α**	57.90 (42.80-72.90)	120.00 (61.30-170.00)	86.60 (39.40-176.00)	73.70 (41.10-92.90)
**Chemokines**	**IL-8**	426.70 (343.40-510.00)	2023.00 (848.80-2500.00)	1383.00 (181.30-2254.00)	630.50 (277.90-1712.00)
	**Eotaxin**	0.54 (0.00-1.08)	0.72 (0.00-23.88)	2.72 (0.00-10.58)	2.92 (0.00-9.73)
	**IP-10**	634.00 (180.00-1088.00)	596.00 (118.00-1095.00)	540.00 (124.00-1298.00)	459.00 (307.00-1131.00)
	**MCP-1**	2395.00 (412.00-4377.00)	2793.00 (151.00-8988.00)	2150.00 (1181.00-4015.00)	2593.00 (1151.00-4724.00)
	**MIP-1α**	23.30 (12.20-34.50)	109.00 (54.00-201.00)	65.10 (27.40-95.20)	35.30 (16.60-98.10)
	**MIP-1β**	1031.00 (537.00-1525.00)	1665.00 (1136.00-2121.00)	1480.00 (854.00-2035.00)	1082.00 (922.00-1929.00)
	**RANTES**	105.00 (0.00-210.00)	423.00 (144.00-2930.00)	739.00 (0.00-1289.00)	0.00 (0.00-165.00)
**Growth factors**	**FGF**	32.64 (28.64-36.64)	40.56 (17.94-62.26)	36.28 (27.62-88.66)	30.08 (15.82-54.53)
	**G-CSF**	1129.00 (596.00-1663.00)	3985.00 (1582.00-6759.00)	2116.00 (1102.00-3599.00)	1400.00 (761.00-3481.00)
	**GM-CSF**	3.78 (2.92-4.64)	10.20 (6.02-11.50)	6.28 (3.86-11.50)	4.56 (0.38-12.90)
	**VEGF**	71.80 (12.80-131.00)	107.00 (48.00-245.00)	167.00 (2.94-391.00)	188.00 (94.20-307.00)
	**PDGF**	14.10 (0.00-28.20)	57.80 (0.00-221.00)	26.40 (9.80-111.00)	18.30 (0.00-52.40)

Function	Analyte	Median (IQR) pg/mL			
		AgB3 pool			
		CE3a	CE3b	CE4/CE5	CTR
Cytokines	IL-1β	78.64 (45.40-111.90)	34.80 (4.58-453.60)	64.32 (28.08-245.10)	37.42 (14.57-156.70)
	IL1rα	12260.00 (11258.00-13263.00)	5325.00 (1891.00-9709.00)	6587.00 (3314.00-11824.00)	8808.00 (5035.00-12043.00)
	IL-2	28.98 (22.52-35.44)	36.72 (13.40-109.00)	45.88 (25.56-134.20)	19.74 (11.53-74.70)
	Il-4	10.42 (6.52-14.32)	16.12 (2.34-33.40)	12.16 (9.22-26.68)	8.00 (4.94-24.00)
	IL-5	171.40 (96.96-245.80)	214.40 (43.38-563.90)	233.50 (149.10-522.20)	187.30 (105.40-404.70)
	IL-6	1101.00 (764.40-1438.00)	1433.00 (21.92-5849.00)	1310.00 (550.40-2714.00)	520.20 (254.90-2695.00)
	IL-7	0.00 (0.00-0.00)	0.00 (0.00-8.94)	17.60 (0.00-28.66)	0.00 (0.00-22.58)
	IL-9	47.54 (24.36-70.72)	53.68 (12.72-140.80)	111.50 (48.22-128.50)	41.48 (11.64-123.90)
	IL-10	17.82 (13.72-21.92)	6.56 (1.56-54.44)	20.16 (8.90-50.18)	8.36 (5.38-37.69)
	IL-12	17.26 (17.08-17.44)	30.04 (4.40-70.28)	28.84 (11.46-59.58)	25.36 (11.28-50.40)
	IL-13	1.36 (0.36-2.36)	2.08 (1.00-4.64)	5.04 (0.84-11.12)	1.22 (0.00-6.91)
	IL-15	539.00 (365.30-712.70)	482.20 (43.04-967.80)	600.60 (467.90-916.10)	385.70 (186.20-1019.00)
	IL-17	54.00 (35.24-72.76)	77.68 (9.62-175.10)	69.00 (37.72-140.70)	28.16 (17.01-120.00)
	IFN-γ	238.00 (187.00-289.00)	140.00 (69.70-162.00)	144.00 (96.10-260.00)	191.00 (121.00-266.00)
	TNF-α	146.00 (108.00-184.00)	221.00 (34.70-545.00)	199.00 (77.90-459.00)	102.00 (58.40-323.00)
Chemokines	IL-8	4599.00 (1295.00-7903.00)	3485.00 (722.40-16786.00)	2196.00 (1408.00-8778.00)	1020.00 (433.70-44187.00)
	Eotaxin	1.10 (0.00-2.20)	0.00 (0.00-33.84)	3.12 (0.00-11.00)	0.00 (0.00-9.13)
	IP-10	1351.00 (950.00-1752.00)	355.00 (32.00-4683.00)	1500.00 (327.00-4032.00)	1268.00 (724.00-2608.00)
	MCP-1	3581.00 (3284.00-3878.00)	1065.00 (309.00-2876.00)	3876.00 (2600.00-7270.00)	5297.00 (1933.00-11184.00)
	MIP-1α	91.20 (41.30-141.00)	539.00 (9.84-1195.00)	154.00 (85.60-1032.00)	83.90 (41.50-970.00)
	MIP-1β	1798.00 (1122.00-2473.00)	2981.00 (173.00-4919.00)	2341.00 (1449.00-9119.00)	1717.00 (1065.00-8181.00)
	RANTES	116.00 (0.00-232.00)	465.00 (215.00-1212.00	773.00 (143.00-1066.00	184.00 (0.00-570.00)
Growth factors	FGF	78.82 (55.00-102.60)	103.60 (5.16-198.20)	71.36 (50.32-185.40)	43.12 (32.01-152.00)
	G-CSF	3414 (1558.00-5270.00)	17999.00 (425.00-24523.00)	5057.00 (3035.00-21117.00)	3022.00 (1787.00-20651.00)
	GM-CSF	8.76 (5.72-11.80)	15.20 (1.12-31.90)	13.20 (10.00-27.50)	9.40 (4.00-30.30)
	VEGF	298.00 (211.00-384.00)	285.00 (70.70-447.00)	395.00 (244.00-631.00)	237.00 (122.00-637.00)
	PDGF	47.40 (39.00-55.80)	34.30 (16.40-293.00)	141.00 (45.50-225.00)	67.40 (1.30-167.00)

Function	Analyte	Median (IQR) pg/mL			
		AgB4 pool			
		**CE3a**	**CE3b**	**CE4/CE5**	**CTR**
**Cytokines**	**IL-1β**	509.80 (56.76-962.80)	41.48 (17.44-249.20)	96.24 (18.56-219.70	149.20 (38.38-367.90)
	**IL1rα**	16959.00(10579.00-23338.00)	7267.00 (4189.00-12460.00)	6710.00 (4268.00-13765.00)	10248.00 (5775.00-19696.00)
	**IL-2**	150.40 (25.56-275.20)	33.44 (29.58-111.50)	68.56 (26.84-150.00)	68.68 (27.73-103.10)
	**IL-4**	27.10 (9.08-45.12)	15.04 (6.58-33.52)	14.16 (8.38-34.02)	27.14 (9.03-35.31)
	**IL-5**	458.40 (150.60-766.30)	243.80 (155.70-545.30)	351.70 (160.00-700.00)	460.70 (200.10-753.60)
	**IL-6**	3003.00 (876.20-5130.00)	783.30 (291.40-6520.00)	1811.00 (647.70-7682.00)	2756.00 (582.80-5523.00)
	**IL-7**	8.78 (0.00-17.56)	0.00 (0.00-0.00)	23.24 (8.86-30.54)	6.58 (0.00-20.01)
	**IL-9**	121.40 (97.48-145.20)	109.30 (68.52-162.60)	120.80 (54.04-286.40)	154.70 (73.76-238.20)
	**IL-10**	54.02 (26.64-81.40)	17.76 (6.76-88.04)	28.96 (9.76-202.80)	84.78 (12.68-256.80)
	**IL-12**	44.96 (17.08-72.84)	22.92 (5.34-72.74)	30.76 (18.30-78.34)	72.04 (34.78-78.68)
	**IL-13**	25.62 (0.72-50.52)	2.88 (1.04-5.36)	6.72 (1.68-15.76)	1.50 (0.74-9.23)
	**IL-15**	1092.00 (449.20-1734.00)	431.00 (177.00-911.90)	690.50 (473.70-1229.00)	757.50 (587.30-1166.00)
	**IL-17**	177.10 (43.12-311.00)	50.00 (20.46-167.20)	67.04 (40.30-190.50)	135.10 (32.81-185.10)
	**IFN-γ**	301.00 (236.00-366.00)	207.00 (97.10-241.00)	205.00 (129.00-294.00)	224.00 (148.00-422.00)
	**TNF-α**	351.00 (115.00-588.00)	181.00 (116.00-627.00)	179.00 (128.00-937.00)	348.00 (112.00-781.00)
**Chemokines**	**IL-8**	29698.00 (3428.00-55968.00)	11321.00 (3096.00-29831.00)	4537.00 (1479.00-19501.00)	8944.00 (2125.00-30103.00)
	**Eotaxin**	7.58 (1.36-13.80)	0.00 (0.00-20.28)	0.00 (0.00-8.20)	0.00 (0.00-10.18)
	**IP-10**	1361.00 (581.00-2140.00)	1610.00 (182.00-2463.00)	1105.00 (524.00-3796.00)	1278.00 (166.00-4305.00)
	**MCP-1**	5402.00 (4731.00-6073.00)	3906.00 (465.00-9736.00)	3604.00 (2647.00-5178.00)	3984.00 (3098.00-9766.00)
	**MIP-1α**	351.00 (78.40-625.00)	165.00 (95.50-1340.00)	229.00 (103.00-1143.00)	736.00 (127.00-1379.00)
	**MIP-1β**	5294.00 (1388.00-9199.00)	2216.00 (1277.00-17278.00)	2768.00 (2027.00-31276.00)	3789.00 (2144.00-25479.00)
	**RANTES**	1687.00 (559.00-2815.00)	1972.00 (487.00-2937.00)	1031.00 (98.20-2084.00)	1312.00 (231.00-2189.00)
**Growth factors**	**FGF**	181.90 (70.16-293.70)	46.20 (28.46-213.60)	99.00 (57.30-214.70)	160.60 (51.36-246.80)
	**G-CSF**	10242.00 (2578.00-17906.00)	4901.00 (3056.00-25880.00)	7097.00 (3389.00-21188.00)	13402.00 (4447.00-27800.00)
	**GM-CSF**	22.40 (8.12-36.60)	11.50 (7.32-32.00)	17.70 (10.00-33.20)	25.30 (7.25-40.70)
	**VEGF**	637.00 (284.00-990.00)	289.00 (172.00-494.00)	384.00 (248.00-873.00)	568.00 (313.00-684.00)
	**PDGF**	373.00 (139.00-607.00)	197.00 (32.40-390.00)	141.00 (43.10-252.00)	272.00 (11.60-430.00)

Function	Analyte	Median (IQR) pg/mL			
		AgB5 pool			
		CE3a	CE3b	CE4/CE5	CTR
Cytokines	**IL-1β**	7.04 (2.52-11.56)	26.60 (7.32-27.64)	14.08 (4.34-45.36)	34.86 (3.64-78.82)
	**IL1rα**	2452.00 (1076.00-3827.00)	3466.00 (1555.00-4234.00)	2371.00 (822.50-4079.00)	4429.00 (661.50-9563.00)
	**IL-2**	4.60 (0.00-9.20)	16.56 (8.32-37.40)	16.24 (6.52-40.64)	16.34 (1.55-49.33)
	**Il-4**	1.46 (0.12-2.80)	6.72 (2.00-19.08)	3.76 (1.20-7.12)	6.90 (0.94-19.21)
	**IL-5**	38.10 (0.00-76.20)	129 (21.04-238.60)	76.92 (40.86-125.40)	124.30 (36.80-334.90)
	**IL-6**	123.60 (7.44-239.80)	152.20 (120.00-1424.00)	190.00 (45.08-369.80)	375.10 (39.15-1488.00)
	**IL-7**	2.30 (0.00-4.60)	0.00 (0.00-11.76)	10.68 (0.00-17.34)	9.94 (0.00-20.70)
	**IL-9**	32.58 (3.68-61.48)	21.12 (0.00-86.76)	52.92 (25.14-93.64)	53.38 (5.39-97.06)
	**IL-10**	9.46 (2.12-16.80)	4.04 (0.00-5.24)	5.08 (2.48-11.32)	8.42 (1.85-26.69)
	**IL-12**	2.32 (0.00- 4.64)	0.00 (0.00-33.28)	7.20 (1.62-21.28)	17.56 (9.71-47.87)
	**IL-13**	0.44 (0.00-0.88)	1.08 (0.00-2.08)	1.44 (0.00-3.24)	0.72 (0.00-2.71)
	**IL-15**	100.50 (0.00-201.10)	108.10 (48.72-439.60)	176.80 (0.00-378.50)	268.10 (71.46-855.90)
	**IL-17**	8.08 (4.60-11.56)	19.60 (3.08-80.12)	20.20 (4.78-35.24)	30.58 (0.86-88.67)
	**IFN-γ**	48.20 (8.68-87.60)	77.80 (31.40-216.00)	67.60 (36.90-143.00)	111.00 (4.83-251.00)
	**TNF-α**	37.20 (5.00-69.40)	77.60 (29.80-212.00)	61.00 (19.30-92.90)	99.50 (9.46-268.00)
Chemokines	**IL-8**	916.2 (0.00-1832.00)	1258.00 (1043.00-5332.00)	822.80 (228.90-1342.00)	1033.00 (249.70-5377.00)
	**Eotaxin**	6.96 (0.00-13.92)	1.24 (0.00-21.68)	5.92 (2.70-8.28)	2.04 (0.00-8.34)
	**IP-10**	1062.00 (30.10-2093.00)	972.00 (22.60-2172.00)	437.00 (95.90-1711.00)	410.00 (92.60-4656.00)
	**MCP-1**	860.00 (24.20-1696.00)	650.00 (152.00-937.00)	1577.00 (855.00-3562.00)	3260.00 (71.70-6417.00)
	**MIP-1α**	13.80 (1.72-25.80)	71.00 (15.60-1105.00)	48.40 (11.90-120.00)	61.20 (6.98-434.00)
	**MIP-1β**	626.00 (79.40-1172.00)	1366.00 (500.00-4868.00)	1194.00 (511.00-1539.00)	1281.00 (278.00-4553.00)
	**RANTES**	269.00 (142.00-396.00)	0.00 (0.00-2662.00)	468.00 (25.30-710.00)	104.00 (0.00-1058.00)
Growth factors	**FGF**	16.24 (0.00-32.48)	34.68 (32.56-115.20)	28.08 (16.72-64.88)	36.92 (4.66-106.10)
	**G-CSF**	456.00 (35.90-875.00)	2565.00 (726.00-27132.00)	1742.00 (517.00-4300.00)	2476.00 (263.00-12680.00)
	**GM-CSF**	3.24 (0.00-6.48)	7.24 (4.28-17.60)	5.20 (2.92-7.56)	7.02 (1.48-21.30)
	**VEGF**	44.40 (0.00-88.70)	106.00 (63.60-214.00)	150.00 (22.90-335.00)	171.00 (65.00-608.00)
	**PDGF**	263.00 (14.20-511.00)	50.60 (0.00-243.00)	33.00 (8.90-101.00)	73.20 (33.30-140.00)

Function	Analyte	Median (IQR) pg/mL			
		AgB total pool			
		CE3a	CE3b	CE4/CE5	CTR
Cytokines	IL-1β	22.20 (21.28-33.44)	26.28 (14.96-81.64)	60.44 (19.12-390.90)	42.50 (11.42-93.01)
	IL1rα	10868.00 (7551.00-19690.00)	5818.00 (4586.00-9660.00)	7957.00 (3743.00-14239.00)	7930.00 (5078.00-15442.00)
	IL-2	273.30 (9.16-330.20)	101.20 (65.12-134.60)	76.48 (28.08-208.90)	22.94 (11.06-45.92)
	Il-4	13.16 (3.56-16.52)	15.88 (7.36-23.16)	15.96 (7.04-43.84)	10.60 (5.08-16.22)
	IL-5	285.90 (73.12-312.00)	295.00 (127.10-370.20)	298.10 (139.60-920.80)	177.50 (129.40-313.10)
	IL-6	299.80 (258.80-1308.00)	517.70 (281.90-2621.00)	1187.00 (469.70-5970.00)	740.60 (263.50-1376.00)
	IL-7	0.00 (0.00-23.92)	0.00 (0.00-6.00)	14.64 (0.00-35.26)	0.00 (0.00-3.34)
	IL-9	115.50 (11.80-150.90)	53.68 (41.42-148.90)	114.20 (57.96-245.70)	76.42 (32.47-88.21)
	IL-10	9.88 (5.04-17.32)	11.96 (3.66-24.34)	17.60 (5.94-203.20)	15.26 (7.44-22.75)
	IL-12	23.12 (11.00-24.68)	14.12 (3.54-69.26)	35.08 (18.30-111.70)	26.86 (5.27-38.56)
	IL-13	57.32 (0.00-66.40)	9 (4.54-11.80)	9.32 (4.34-15.46)	0.00 (0.00-2.29)
	IL-15	446.70 (261.80-719.90)	412.00 (179.80-761.70)	637.00 (352.00-1485.00)	453.50 (274.90-658.00)
	IL-17	61.20 (14.88-65.48)	48.16 (25.58-103.60)	62.96 (30.64-245.70)	40.52 (16.84-83.69)
	IFN-γ	171.00 (171.00-277.00)	151.00 (97.00-296.00)	177.00 (134.00-286.00)	167.00 (134.00-288.00)
	TNF-α	205.00 (45.10-226.00)	205.00 (95.80-325.00)	129.00 (88.20-1059.00)	116.00 (57.90-292.00)
Chemokines	IL-8	10219.00 (926.00-21943.00)	5604.00 (2857.00-10421.00)	2810.00 (993.90-32861.00)	1359.00 (636.60-9102.00)
	Eotaxin	2.60 (0.00-14.44)	0.00 (0.00-12.00)	3.72 (0.28-11.44)	0.74 (0.00-13.59)
	IP-10	852.00 (845.00-4763.00)	711.00 (573.00-10566.00)	4351.00 (1038.00-9206.00)	1255.00 (676.00-7529.00)
	MCP-1	2438.00 (2078.00-8340.00)	4660.00 (705.00-13417.00)	3934.00 (2936.00-5312.00)	3099.00 (2178.00-10962.00)
	MIP-1α	201.00 (25.70-1057.00)	282.00 (88.70-852.00)	234.00 (79.10-1136.00)	97.10 (70.40-253.00)
	MIP-1β	4115.00 (799.00-25910.00)	2400.00 (1239.00-4588.00)	2902.00 (1879.00-20587.00)	1991.00 (1708.00-3211.00)
	RANTES	4051.00 (0.00-5295.00)	315.00 (109.00-2673.00)	777.00 (309.00-1562.00)	517.00 (95.10-780.00)
Growth factors	FGF	71.00 (28.64-79.88)	55.84 (18.80-141.10)	87.80 (52.48-265.10)	57.86 (34.05-83.10)
	G-CSF	5047.00 (1159.00-11543.00)	9649.00 (2485.00-17850.00)	6518.00 (3129.00-22359.00)	3168.00 (2085.00-7877.00)
	GM-CSF	15.10 (4.08-18.40)	19.20 (7.96-23.50)	18.90 (9.64-39.40)	9.08 (1.93-17.90)
	VEGF	333.00 (142.00-376.00)	319.00 (204.00-403.00)	526.00 (186.00-1099.00)	255.00 (174.00-458.00)
	PDGF	294.00 (0.00-545.00)	99.20 (38.80-349.00)	145.00 (40.50-348.00)	58.60 (1.54-112.00)

Function	Analyte	Median (IQR) pg/mL			
		Native AgB			
		CE3a	CE3b	CE4/CE5	CTR
Cytokines	**IL-1β**	17.96 (0.00-178.20)	5.28 (0.24-15.52)	1.28 (0.24-18.04)	0.50 (0.04-485.10)
	**IL1rα**	10993.00 (0.00-15255.00)	755.20 (358.20-6995.00)	880.60 (206.40-1801.00)	454.20 (135.90-6922.00)
	**IL-2**	397.50 (0.00-546.70)	85.04 (29.64-434.50)	40.64 (10.30-373.90)	0.84 (0.00-172.20)
	**Il-4**	5.72 (0.12-30.20)	9.92 (0.00-19.44)	2.24 (0.34-21.18)	0.48 (0.25-36.04)
	**IL-5**	54.52 (0.00-617.50)	226.20 (30.42-242.40)	52.32 (12.82-389.40)	17.08 (0.00-780.00)
	**IL-6**	76.32 (0.40-3355.00)	9.08 (2.58-1167.00)	32.00 (2.64-643.70)	2.72 (0.00-11058.00)
	**IL-7**	0.00 (0.00-16.60)	0.00 (0.00-0.00)	6.32 (0.00-15.20)	12.84 (0.03-30.49)
	**IL-9**	25.08 (9.60-233.10)	69.88 (8.12-89.16)	50.36 (23.96-79.32)	20.72 (3.56-335.50)
	**IL-10**	2.16 (0.00-24.16)	3.16 (0.30-8.08)	1.12 (0.00-19.90)	1.06 (0.24-326.90)
	**IL-12**	5.88 (0.00-68.40)	20.96 (0.00-43.80)	11.96 (0.00-47.50)	5.50 (0.00-109.40)
	**IL-13**	56.60 (0.00-124.20)	6.88 (2.02-37.58)	2.46 (0.80-17.27)	0.28 (0.00-3.09)
	**IL-15**	440.00 (0.00-797.30)	231.00 (0.00-514.30)	148.80 (0.00-751.20)	13.78 (0.00-992.80)
	**IL-17**	19.68 (1.16-148.60)	27.08 (0.00-74.72)	7.56 (0.58-84.74)	3.50 (0.29-212.40)
	**IFN-γ**	126.00 (0.00-214.00)	91.10 (36.50-153.00)	63.30 (5.70-148.00)	10.20 (5.14-183.00)
	**TNF-α**	124.00 (0.00-779.00)	148.00 (16.20-288.00)	28.60 (0.64-174.00)	10.50 (5.13-2490.00)
Chemokines	**IL-8**	13445.00 (0.00-44554.00)	2382.00 (1333.00-22958.00)	676.30 (232.00-19631.00)	740.20 (47.21-20345.00)
	**Eotaxin**	5.72 (4.72-10.72)	0.00 (0.00-0.00)	2.36 (0.00-7.36)	11.72 (2.01-34.35)
	**IP-10**	54.50 (0.00-842.00)	957.00 (0.00-1655.00)	127.00 (21.10-891.00)	144.00 (9.55-447.00)
	**MCP-1**	1359.00 (0.00-10920.00)	755.00 (4.02-12230.00)	884.00 (143.00-3520.00)	109.00 (78.00-1340.00)
	**MIP-1α**	85.90 (0.00-1285.00)	167.00 (2.54-872.00)	34.10 (7.48-781.00)	1.28 (0.94-1113.00)
	**MIP-1β**	1827.00 (0.00-31964.00)	1376.00 (63.90-3692.00)	932.00 (216.00-2720.00)	94.50 (27.00-23924.00)
	**RANTES**	573.00 (0.00-9130.00)	160.00 (0.00-610.00)	188.00 (0.00-668.00)	359.00 (38.80-1919.00)
Growth factors	**FGF**	44.32 (0.00-155.50)	32.56 (5.66-97.96)	18.36 (3.86-112.50)	0.68 (0.00-209.70)
	**G-CSF**	3245.00 (0.00-12731.00)	6377.00 (130.00-17607.00)	1495.00 (238.00-13341.00)	80.30 (18.60-15305.00)
	**GM-CSF**	9.24 (0.20-41.60)	19.90 (2.80-20.50)	2.84 (1.14-44.10)	1.20 (0.23-41.20)
	**VEGF**	95.20 (85.20-667.00)	193.00 (24.60-399.00)	126.00 (0.00-518.00)	15.00 (1.98-618.00)
	**PDGF**	115.00 (0.00-519.00)	64.90 (0.00-89.70)	23.70 (0.00-101.00)	39.90 (6.61-436.00)

Footnotes: AgB: antigen B; IQR: interquartile range; analyte concentrations were determined by luminex. IL: interleukin; ra: receptor antagonist; FGF: fibroblast growth factor; G-CSF: granulocyte colony-stimulating factor; GM-CSF: granulocyte-macrophage colony-stimulating factor; IFN: Interferon; IP: Interferon gamma-induced protein; MCP: monocyte chemoattractant protein; MIP: macrophage inflammatory protein; PDGF: platelet-derived growth factor; RANTES: regulated on activation, normal T cell expressed and secreted; TNF: tumor necrosis factor; VEGF: vascular endothelial growth factor.
