# Supplementary figures and images for: Stage-Specific Immune Responses to AgB T-Peptides in Patients with Cystic Echinococcosis
Source: Infect Dis Rep. 2025 May 7;17(3):51. doi: 10.3390/idr17030051 (PMC12101248; doi:10.3390/idr17030051)

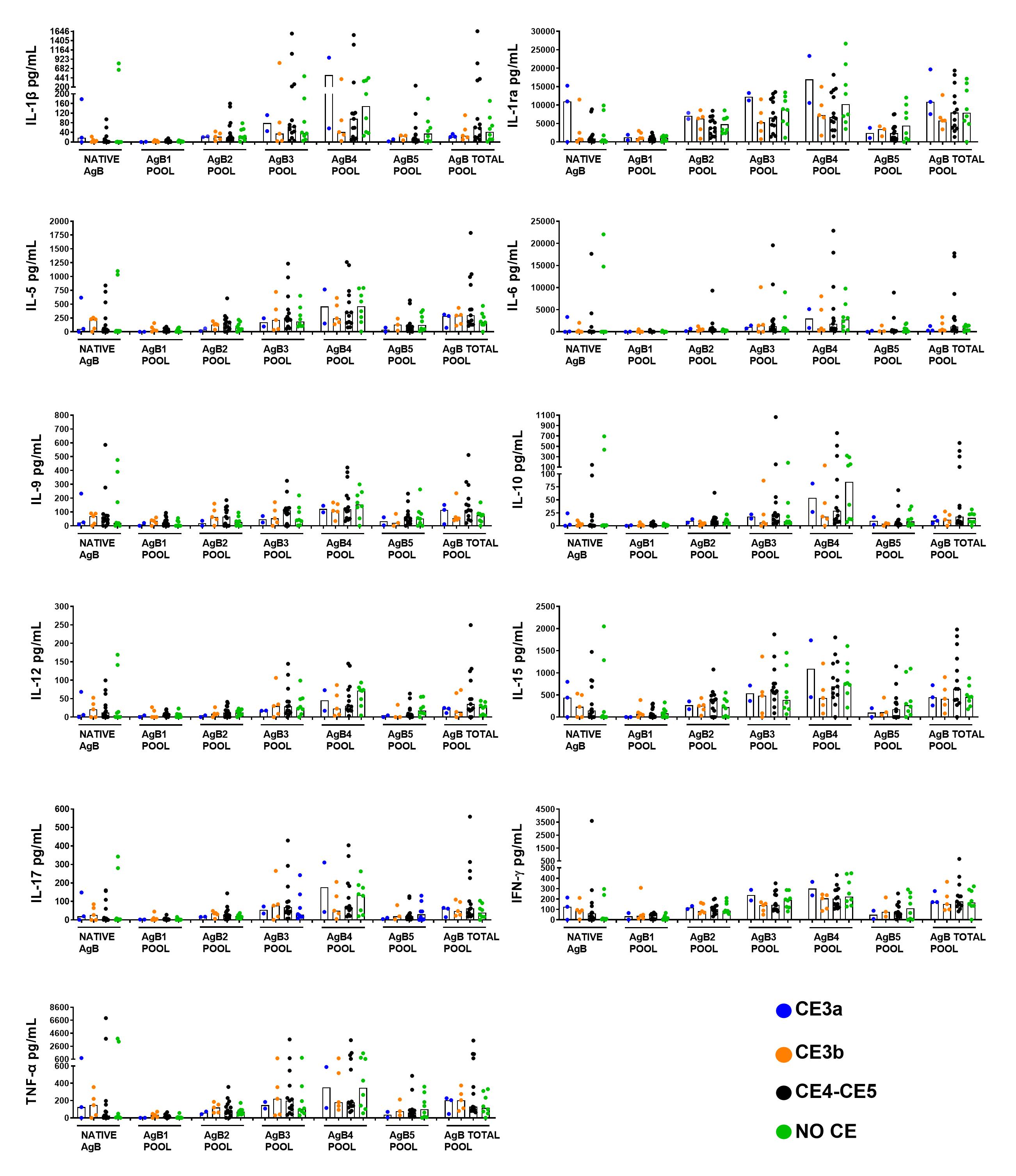

Supplement: Supplementary file 1 [file idr-17-00051-s001.zip › Figure S1.jpg]

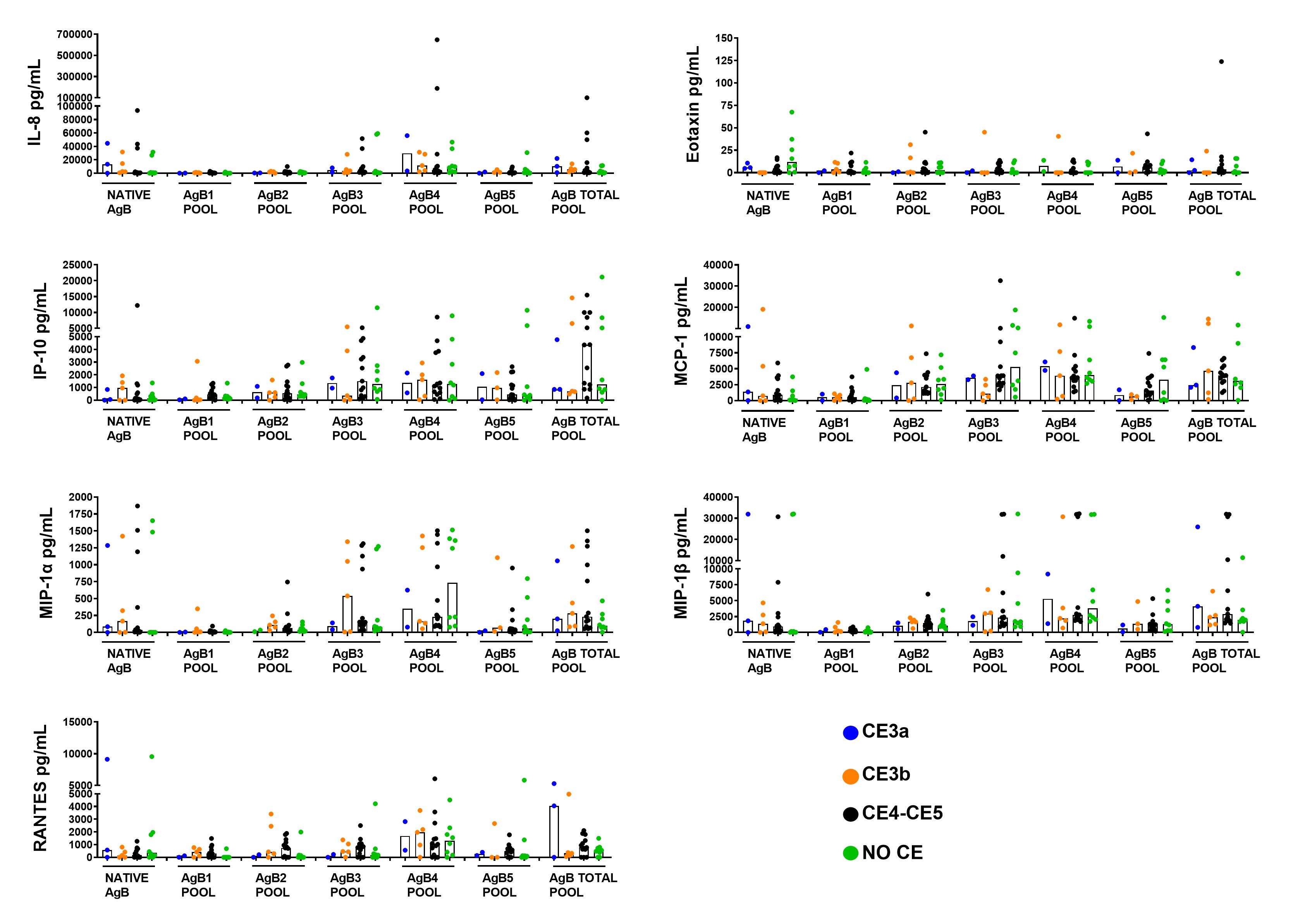

Supplement: Supplementary file 1 [file idr-17-00051-s001.zip › Figure S2.jpg]

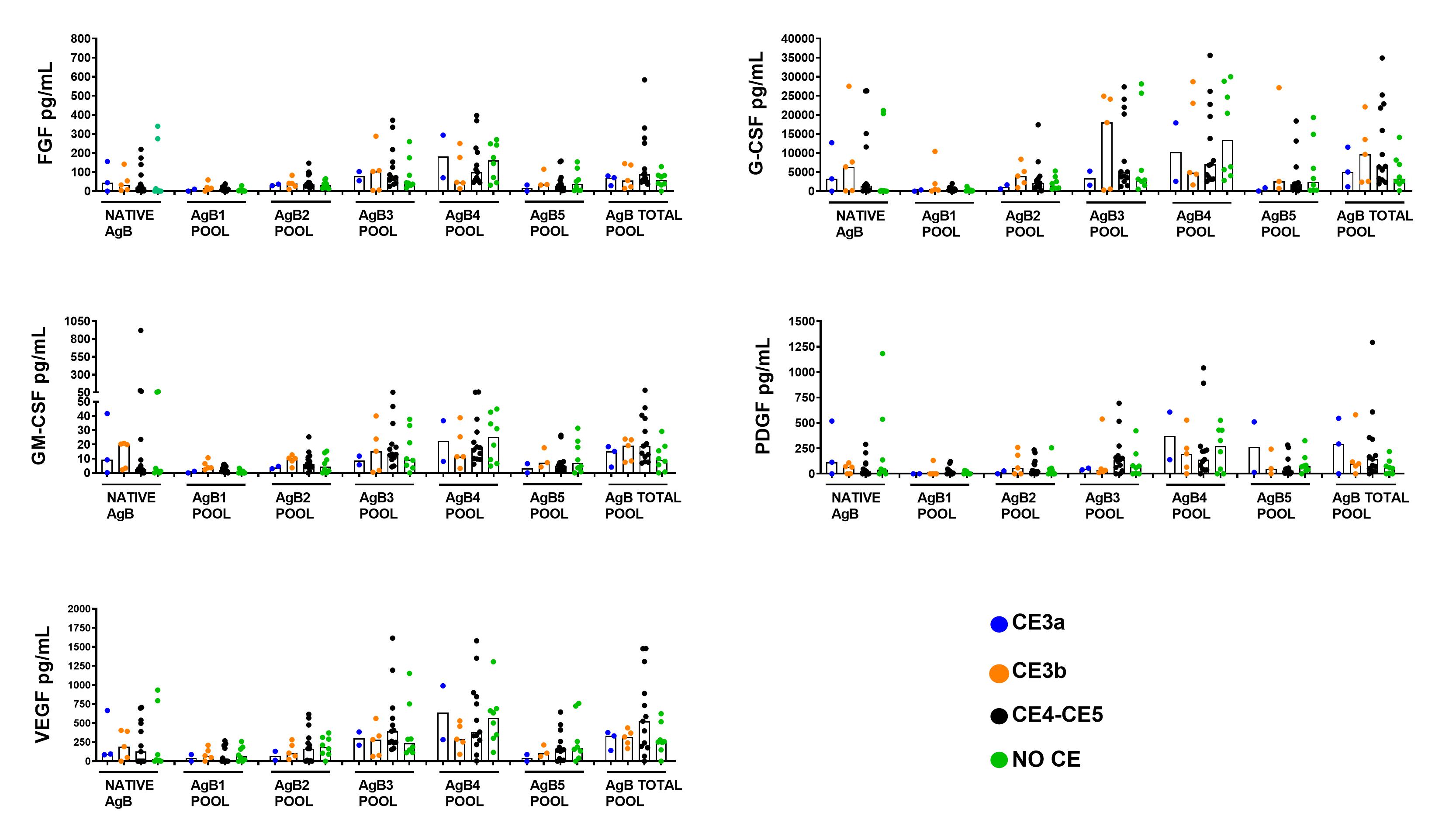

Supplement: Supplementary file 1 [file idr-17-00051-s001.zip › Figure S3.jpg]
